# Supplementary material for: Epidemiology of thalassemia among the hill tribe population in Thailand
Source: PLoS One. 2021 Feb 11;16(2):e0246736. doi: 10.1371/journal.pone.0246736 (PMC7877639; doi:10.1371/journal.pone.0246736)
Supplement: S1 Appendix — (PDF) [file pone.0246736.s001.pdf]

รหัสแบบสอบถาม ___________________

แบบสอบถามโครงการวิจัย

เรื่อง อัตราความชุกของการเป็นพาหะธาลัสซีเมียในชาวเขา

1. อายุ______ ปี

2. เพศ ☐ □ ชาย ☐ □หญิง

3. น้ำหนัก _________ กิโลกรัม ส่วนสูง _________ เซนติเมตร

4. ท่านนับถือศาสนาใด

☐ พุทธ ☐ คริสต์ ☐ อิสลาม ☐ อื่นๆ(โปรดระบุ) ______

5. ท่านเรียนอยู่ชั้นอะไร

☐ ประถมศึกษาปีที่ 4 ☐ ประถมศึกษาปีที่ 5 ☐ ประถมศึกษาปีที่ 6

6. ท่านมีจำนวนสมาชิกในครอบครัว _________ คน เป็นพี่น้องร่วมสายโลหิต (ที่ยังมีชีวิตอยู่) ________ คน

7. คุณพ่อเป็นเผ่าอะไร

☐ อาข่า (อีก้อ) ☐□ ลาหู่ (มูเซอ) ☐□ ลีซู

☐ ม้ง ☐□ เย้า ☐□ กะเหรี่ยง

☐ ไทยใหญ่ ☐□ ลั๊วะ ☐□ ไทยลื้อ

☐ จีนฮ่อ ☐□ อื่นๆ (โปรดระบุ) ____________________

8. คุณแม่เป็นเผ่าอะไร

☐ อาข่า (อีก้อ) ☐□ ลาหู่ (มูเซอ) ☐□ ลีซู

☐ ม้ง ☐□ เย้า ☐□ กะเหรี่ยง

☐ ไทยใหญ่ ☐□ ลั๊วะ ☐□ ไทยลื้อ

☐ จีนฮ่อ ☐□ อื่นๆ (โปรดระบุ) ____________________

9. คุณปู่(พ่อของพ่อ) เผ่าอะไร

☐ อาข่า (อีก้อ) ☐□ ลาหู่ (มูเซอ) ☐□ ลีซู

☐ ม้ง ☐□ เย้า ☐□ กะเหรี่ยง

☐ ไทยใหญ่ ☐□ ลั๊วะ ☐□ ไทยลื้อ

☐ จีนฮ่อ ☐□ อื่นๆ (โปรดระบุ) ____________________

10. คุณย่า (แม่ของพ่อ) เป็นเผ่าอะไร

☐ อาข่า (อีก้อ) ☐□ ลาหู่ (มูเซอ) ☐□ ลีซู

☐ ม้ง ☐□ เย้า ☐□ กะเหรี่ยง

☐ ไทยใหญ่ ☐□ ลั๊วะ ☐□ ไทยลื้อ

☐ จีนฮ่อ ☐□ อื่นๆ (โปรดระบุ) ____________________

11. คุณตา (พ่อของแม่) เป็นเผ่าอะไร

☐ อาข่า (อีก้อ) ☐☐ ลาหู่ (มูเซอ) ☐☐ ลีซู

☐ ม้ง ☐☐ เย้า ☐☐ กะเหรี่ยง

☐ ไทยใหญ่ ☐☐ ลั๊วะ ☐☐ ไทยลื้อ

☐ จีนฮ่อ ☐☐ อื่นๆ (โปรดระบุ) ______________________

12. คุณยาย (แม่ของแม่) เป็นเผ่าอะไร

☐ อาข่า (อีก้อ) ☐☐ ลาหู่ (มูเซอ) ☐☐ ลีซู

☐ ม้ง ☐☐ เย้า ☐☐ กะเหรี่ยง

☐ ไทยใหญ่ ☐☐ ลั๊วะ ☐☐ ไทยลื้อ

☐ จีนฮ่อ ☐☐ อื่นๆ (โปรดระบุ) ______________________

13. ท่านมีโรคประจำตัวหรือไม่

☐☐ ไม่มี

☐ มี ได้แก่ (ตอบได้มากกว่า 1 ข้อ)

☐☐ โรคเบาหวาน ☐☐ ไขมันในเลือดสูง

☐☐ โรคหัวใจ ☐☐ โรคภูมิแพ้

☐ โรคหอบหืด ☐☐ โรคกระเพาะอาหาร

☐☐ โรคกระดูกและข้อ ☐ โรคมะเร็ง

☐☐ อื่นๆ (โปรดระบุ) ______________

14. ท่านเคยมีอาการใด หรือตรวจพบอาการเหล่านี้บ้างหรือไม่ (ตอบได้มากกว่า 1 ข้อ)

☐☐ โลหิตจาง ☐ ซีดเซียว

☐☐ อ่อนล้า/อ่อนเพลีย ☐ มีเลือดปนในปัสสาวะ

☐☐ หายใจสั้น ☐ ☐ ภาวะดีซ่าน/อาการตัวเหลือง

☐☐ ม้ามโต ☐ ตับโต

15. ท่านเคยได้รับเลือดเพราะภาวะซีด

☐☐ ไม่เคย ☐☐ เคย

16. พี่หรือน้องของท่าน เคยได้รับเลือดเพราะภาวะซีด

☐ ไม่มี ☐ มี (โปรดระบุ) ______________

17. บุคคลในเครือญาติของท่าน เคยได้รับเลือดเพราะภาวะซีด

☐☐ ไม่มี ☐☐ มี (โปรดระบุ) ______________

18. ท่านเคยกินยาบำรุงเลือด หรือเสริมธาตุเหล็กหรือไม่

☐ ไม่เคย ☐ เคย
