# Supplementary material for: Epidemiology of thalassemia among the hill tribe population in Thailand
Source: PLoS One. 2021 Feb 11;16(2):e0246736. doi: 10.1371/journal.pone.0246736 (PMC7877639; doi:10.1371/journal.pone.0246736)
Supplement: S2 Appendix — (PDF) [file pone.0246736.s002.pdf]

CODE___________________

# Questionnaire

1. Age______ year

2. Sex ☐ Male ☐ Female

3. Weight __________kg Height __________cm

4. Religion

   ☐ Buddhist ☐ Christian ☐ Islam ☐ Othe (Specify) _______

5. Education

   ☐Grade 4 ☐ Grade 5 ☐ Grade 6

6. Number of family member __________ person(s), number of alive siblinges ________ person(s)

7. Father's tribe

| | | |
|---|---|---|
| ☐ Akha | ☐ Lahu | ☐ Lisu |
| ☐ Hmong | ☐ Yao | ☐ Karen |
| ☐ Thai-Yai | ☐ Lua | ☐ Thai-Lure |
| ☐ Chinese | ☐ Other (Specify) | |

8. Mother's tribe

| | | |
|---|---|---|
| ☐ Akha | ☐ Lahu | ☐ Lisu |
| ☐ Hmong | ☐ Yao | ☐ Karen |
| ☐ Thai-Yai | ☐ Lua | ☐ Thai-Lure |
| ☐ Chinese | ☐ Other (Specify) | |

9. Grandfather (Father of father) tribe

| | | |
|---|---|---|
| ☐ Akha | ☐ Lahu | ☐ Lisu |
| ☐ Hmong | ☐ Yao | ☐ Karen |
| ☐ Thai-Yai | ☐ Lua | ☐ Thai-Lure |
| ☐ Chinese | ☐ Other (Specify) | |

10. Grandmother (Mother of father) tribe

| | | |
|---|---|---|
| ☐ Akha | ☐ Lahu | ☐ Lisu |
| ☐ Hmong | ☐ Yao | ☐ Karen |
| ☐ Thai-Yai | ☐ Lua | ☐ Thai-Lure |
| ☐ Chinese | ☐ Other (Specify) | |

11. Grandfather (Father of mother) tribe

☐ Akha ☐ Lahu ☐ Lisu

☐ Hmong ☐ Yao ☐ Karen

☐ Thai-Yai ☐ Lua ☐ Thai-Lure

☐ Chinese ☐ Other (Specify)

12. Grandmother (Mother of mother) tribe

☐ Akha ☐ Lahu ☐ Lisu

☐ Hmong ☐ Yao ☐ Karen

☐ Thai-Yai ☐ Lua ☐ Thai-Lure

☐ Chinese ☐ Other (Specify)

13. Medical illness

☐ No

☐ Yes (Possible to choose more than one option)

☐ Diabetes ☐Hyperlipidemia

☐ Heart disease ☐Allergic

☐ Asthma ☐ Gastritis

☐ Joint disease ☐ cancer

☐ Other (specify............)

14. Have you had any diagnosis in the following disease (Possible to choose more than one option)

☐ Anemia ☐ Pale

☐ Weak ☐ มีเลือดปนในปัสสาวะ

☐ Short breathing ☐ Jaundice

☐ Splenomegaly ☐ Enlarged liver

15. Have you had blood transfusion ?

☐ No ☐ Yes (Specify) _______________

16. Do you have any siblings who took anemia disease or folic acid?

☐ No ☐ Yes (Specify) _______________

17. Do you have any relative who took anemia disease or folic acid?

☐ No ☐ Yes (Specify) _______________

18. Have you ever took anemia disease or folic acid?

☐ No ☐ Yes
