# Supplementary material for: Epidemiology of thalassemia among the hill tribe population in Thailand
Source: PLoS One. 2021 Feb 11;16(2):e0246736. doi: 10.1371/journal.pone.0246736 (PMC7877639; doi:10.1371/journal.pone.0246736)
Supplement: Attachment — Submitted filename: Journal_Requirements.docx [file pone.0246736.s004.docx]

Journal Requirements:

When submitting your revision, we need you to address these additional requirements.

1. Please ensure that your manuscript meets PLOS ONE's style requirements, including those for file naming. The PLOS ONE style templates can be found at

<https://journals.plos.org/plosone/s/file?id=wjVg/PLOSOne_formatting_sample_main_body.pdf> and

<https://journals.plos.org/plosone/s/file?id=ba62/PLOSOne_formatting_sample_title_authors_affiliations.pdf>

 : Thank you, I have checked to ensure that the journal requirements are met.

2. Please include additional information regarding the questionnaire used in the study and ensure that you have provided sufficient details that others could replicate the analyses. For instance, if you developed a questionnaire as part of this study and it is not under a copyright more restrictive than CC-BY, please include a copy, in both the original language and English, as Supporting Information.

: We added that both Thai and English versions of the questionnaire that we developed for this particular study were used.

3. Please state in your methods section the participant recruitment date.

: The information and method of selection is described in the “***Data collection procedure***” section. In brief, a simple random method was used to select the schools, and all students graded 4-6 in selected schools were eligible and invited to participate in the study after obtaining consent from their parents.

4. We note you have included a table to which you do not refer in the text of your manuscript. Please ensure that you refer to Table 7 in your text; if accepted, production will need this reference to link the reader to the Table.

: Thank you, this has been clarified on page 6.

**Comments to the Author**

Reviewer #1: The manuscript by Tawatchai Apidechkul et al. “Epi-genetics of thalassemia among the hill tribe population in Thailand” presents results of a genetic screening, aiming to estimate the prevalence of various forms of thalassemia among the six main hill tribe (minority) populations in Thailand. The issue of management of hemoglobinopathies patients is still apparent despite attempts to be controlled with prenatal testing and it is even nowadays one of the most common genetic diseases. The prevalence of all forms of thalassemia is high in countries with diverse ethnicity, high immigrant rate and in minority societies where consanguineous marriages are widely in practice. The early onset of the disease (few months after birth) and the application of expensive therapeutic protocols, including blood transfusions and chelating agents, adds an unendurable financial burden to healthcare systems.
This study appears to have been carefully performed and conclusions are relevant to the main scope of the study, the mandatory establishment of genetic counseling and prenatal diagnosis for hemoglobin disorders, especially in minority societies such as the hill tribe population in Thailand.

However, there are a number of major issues in the manuscript that need to be addressed before resubmission of revised version:

1) The term “epigenetics” in the title is irrelevant to the subject of this study. Epigenetics refers to biochemical alterations of the histone proteins or genome (DNA) or non-coding RNAs that differentially regulate gene expression levels.

: Thank you for the comment. As my training background is in “epidemiology”, which is ***the study and analysis of the distribution, patterns and determinants of health and disease conditions in defined populations***, and our work focuses on the genetics of thalassemia by using PCR and HPLC, we used the term of “Epi-genetics”.

However, the reviewer did not provide alternate phrasing, and we have changed the title to “Epidemiology of thalassemia among the hill tribe population in Thailand: a cross sectional study”, which seems to well reflect the content and STROBE guideline.

2) The manuscript needs an extensive grammatical and syntactical editing. In its present form does not meet the criteria for publishing.

: The manuscript has been revised twice by the American Journal Experts with the code **07A3-ECE2-5B78-98D6-6F9F** .

we hope that the language has been improved.

3) The authors need to be as precise as possible about how they did the genetic analysis

: Thank you for the comment. We have added detail to the methods section.

a)The equation in the paragraph “sample size”, although its reference is given, its terms are not (even briefly) described.

: Thank you for the comment; this has been clarified.

b) The whole section of “materials and methods” is insufficient. The lab tests must be described and results obtained by each method performed, must be stated. Differences detected between lab methods and presented in the discussion section, must be explained and substantiated.

: The details of the laboratory methods have been added to the “Laboratory test” section. The results in each step or method are clearly presented in Tables 4-7, including elaboration of the main findings in each section.

: In the discussion, we added to present the overall results and comparisons with other studies and populations, with possible reasons for differences. We also added information per your comments.

4) A figure presenting α-type and β-type globin genes with specific mutations highlighted from the study should accompany the manuscript to support the text and offer to the audience a better understanding of the data presented.

: Relevant information has been added to the introduction section (first paragraph)

5) The discussion section is confusing. Authors display results of the study but significant and non-significant data are not distinguished, hereditary risk factors for the next generations are not clearly defined, the prognostic value of their results is not supported.

: Thank you for the comment. However, the main aim of the study was to estimate the prevalence and the major form of thalassemia in each specific group of population, including sex and tribe. Therefore, for the main result of the study, we used the laboratory findings to present the situation of thalassemia among the hill tribe, which has never been reported. This is basic and pioneering scientific information regarding thalassemia in these populations. Detecting the risk factors for the next generation was not the main goal and is not to common because thalassemia is genetic-related disease.

We use the term of ‘epidemiology’ to refer to description (Descriptive epidemiology) the characteristics of the person (sex and tribe) and the proportion of different forms of thalassemia, which is a descriptive epidemiology focus.

However, in Tables 4 and 5, the chi-square test is presented to detect differences in the proportion on the OF and DCIP results among patients with different characteristics. The results indicate that females are more likely to have thalassemia than males and that those whose grandfather (father of mother) and grandmother (mother of mother) are of the same tribe were more likely to have thalassemia than those whose grandfather (father of mother) and grandmother (mother of mother) are from different tribes.

Moreover, α-thalassemia 1 was found at a higher rate in females than males, though β-thalassemia was higher among males than females.

We have heavily reviewed all the literature of different sources of medical papers, but unfortunately there is no articles regarding these points.

6. PLOS authors have the option to publish the peer review history of their article ([what does this mean?](https://journals.plos.org/plosone/s/editorial-and-peer-review-process#loc-peer-review-history)). If published, this will include your full peer review and any attached files.

Kind regards,

TK

Assist Prof. Dr. Tawatchai Apidechkul, MSc (Infectious Epidemiology), PhD (Epidemiology)

Deputy Dean, School of Health Science, MFU

Director, Center of Excellence of Hill Tribe Health Research, WHO-CC

Former Hubert H Humphrey Fellow (2013-2014), Emory University

Global Health Delivery Intensive (Harvard School of Public Health)
