# Supplementary material for: Epidemiology of thalassemia among the hill tribe population in Thailand
Source: PLoS One. 2021 Feb 11;16(2):e0246736. doi: 10.1371/journal.pone.0246736 (PMC7877639; doi:10.1371/journal.pone.0246736)
Supplement: Attachment — Submitted filename: Response to reviewers.docx [file pone.0246736.s005.docx]

Response to reviewers’ comments

Dear editor and reviewers,

In this revised version we have revised and confirmed in whole section of laboratory results with consulting experts in the field. We also made more analysis according to the comment of reviewer no.4. We did improvement in telling the story in whole text particularly in first paragraph, and many paragraphs in the method and results sections. It’s also improved by the native speakers to make sure that it could be good enough for the journal.

I can confirm that all the points suggested from revisers have been carefully improved.

I do very hope very hope that you happy in this version.

TK

Reviewer #1: • The title of the study absolutely suits the text.

: Thank you.

• The manuscript has been edited by an expert as authors claim, however, the text flow is tedious, specific points are still difficult for native speakers to follow, the analysis of the results are greatly extended, but with little necessity. The above mentioned issues reduce the readership for the manuscript and does not meet the criteria for publishing in PLOS ONE journal. Nevertheless, genetic analysis of existing minorities worldwide should gain attention due to high frequencies of pathological alleles observed among them.

: The whole paper has been revised and improved by both authors, and the native speakers.

: We thank you for your comment here. Even it is very difficult for us to demonstrate an excellent English, but after carful revisions many times, we do very hope that you will happy in this version.

• Carriers of pathological thalassemia alleles, should be identified as heterozygotes or double heterozygotes in case of the co-existence of two distinct alleles for thalassemia traits.

: Thank you for the great comment. We have revised all terminologies and presented only essential forms in the whole text. However, if I did not make clear to response your comment, please clarify more. Thank you so much.

• In materials and methods the research outline should be presented clearly. The questionnaires cannot be considered as “research instruments”.

: Thank you, we agree with and it’s deleted. We have added laboratory procedures.

• A list of abbreviations is needed.

: We do not sure that the style of Ploseone allows us to present a list of abbreviation separately. However, we have double-checked all abbreviations are placed properly in whole text.

• It would be more interesting to reduce the text in terms of appearing as a “letter to the editor” (than original article), with sharp analysis of results and clear definition of the consequent needs for constant genetic counseling and prenatal diagnosis for hemoglobin disorders in these minorities (hill tribe population of Thailand).

: Thank you for great comment. We have made improvement in whole text particularly in the first paragraph of introduction.

Reviewer #3:

1-I agree with all first reviewer comments, additionally, I have the following comments:

: We have carefully revised and improved all points on the first reviewers’ comments

2-Materials and methods still need additions and clarifications, starting from the manufacturer of all kits used [ KKU-OF reagent KKU-DCIP-Clear reagent kit] instruments as well (e.g what was the brand name of qPCR used and manufacturers, HPLC machine ( Variant II ??) .

: Thank you, all essential information have been placed. Page No.5,

: Variant-II was used for HPLC. Page 5

3-OF and DCIP tests needs more clarifications (as supplement to the paper probably). These tests are not widely spread and the method of action needs to be mentioned. How the authors considered each of these tests results as positive or negative ? any references ? these are manual kits? ,Spectrometer used ? which wavelength ? Please write concise details about each tests to be more.

: Thank you so much for the great comment. We have put extended information relevant to these methods.

: We have added detail of these procedures at page 5 with references.

4- Since the above tests were the screening tests, we need to know the implication of each test. DCIP is mentioned by the authors to measure unstable Hbs, while no such Hb was mentioned in the results. I think HbE ais detected by this kit too.

: Thank you for the comment. At the stage of using either OF or DCIP, we did not intend to make interpretation since there were screening tests.

: Bascially, OF test intends to screen for alpha-thalassemia-1 and beta-thalssemia, while DCIP is screened for Hb E. Then, in our study, after having a positive of either one or both were confirmed by HPLC and PCR before making interpretation.

:If OF+ only, it’s suspected to be alpha-thalassemia and/ or betha-thalssemia, while having DCIP+ only, it’s suspected to be Hb E trait. If having positive in both tests, it could be suspected Hb E with or without alpha-thalassemia, and/or b-thalassemia.

5-Screening was performed using both tests as mentioned but figure -1- states OF or DCIP. Please clarify.

: Sorry for the mistake, it’s correct. Specimens were tested by both tests.

6- Did the authors followed previous guidelines regarding hemoglobinopathies screening and have chosen to organised their plan accordingly ? I am asking because I cant understand why after double positive tests, HPLC test was performed ? Why not after single positive tests for example ?

: Positive to either OF or DCIP, or both OF and DCIP were tested by HPL and PCR.

7- Abbreviations in Fig 1 need standardization. What did the author mean with A2A?

: Thank you, there have been made clarification all abbreviations.

: HbA which is adult hemoglobin, hemoglobin A1 or α_2_β_2_.

: HbA is the most common adult form of hemoglobin and exist as tetramer containing two alpha subunits and two beta subunits (α_2_β_2_)

: HbA2 is a less common adult form of hemoglobin and is composed of two alpha and two delta.

: A2A is a normal variant of hemoglobin A that consists of two alpha and two delta chains (α_2_δ_2_) and is found at low levels in normal human blood.

8-How could the authors identify HbBarts ?

: By doing Hb typing in the HPLC method, those who have presented the low Hb A_2_ level due to having Hb H (β_4_) or Hb Bart’s (γ_4_) which is few of α-globin then the β-globin and γ-globin remain a large portion and catching each other to obtain 4 lines. Then while we doing HPLC, the band of Hb Barts is presented.

9-What were the types of beta genes mutations reported in this study ? The authors have mentioned beta 0 and beta + mutation??

: It could be (β^0^/β) or (β^+^/β)

: However, in our current method, we could not identify in specific genes mustation. Sorry.

10-What was the abnormal Hb type detected (mentioned in Fig 1 ) please ?HPLC usually identify Hb structural variants like HbD, HbE ,HbO ,HbS,.......

: Thank you for the notice.

: A case of abnormal Hb type was detected by HPLC with detail; MCV=73.4lf, positive OF, positive DCIP, 46.0%HbAo, 3.0%HbA2/E, 0.5%HbF, A2A Hb typing with abnormal Hb, and negative for alpha-thalassemia (SEA and Thai deletion).

11-What was the distribution of alfa-thalassemia SEA and Thai deletion among alfa thal minor cases detected?

: Only alpha-thalassemia 1 trait (SEA deletion) was found, data presented in table 6 and 7.

12-In Fig -1- the authors mentioned that qPCR was used to detect alfa thal mutations, while they have reported beta thal mutation and HbE as well (beta structural variant) .There are errors in the results of qPCR, some of them are mentioned in HPLC results too. Please re-arrange the whole Fig 1 .

: Thank you for great comment, we have made revised in whole structure in figure no. 1, and also in the context.

13-i wished to see a comparison of alfa and beta thal mutations reported with surrounded districts or populations, or even the tribes included in the current study.

: It’s reported in the reference no. 21 and 28. There was very few publications on the hill tribe people in Thailand. One more study reported on beta thalassemia among the hill tribe school children (Ref. No. 29).

In conclusion ,
Material and methods needs a good revision, and results accordingly.

: Thank you very much for the great comments. We have revised improved a large scale particularly in extending the content of OF and DCIP including other information as comments.

Reviewer #4: Comments for the author
This paper demonstrated the epidemiology of thalassemia among the hill tribe population in Thailand. The study showed differences in the prevalence of thalassemia and hemoglobinopathies among different tribes. The author also reported the relation between positive OF tests and the same-tribe grandparents. These are interesting findings reflecting the association between consanguineous marriage and thalassemia disease. I have the following comments on this paper:
1.The authors should clarify the remaining 32 patients who have positive OF tests with normal hemoglobin typing by excluding other conditions that may cause false-positive OF tests. It is interesting if the author can further test for alpha-thalassemia-2 (if not, please add this issue in the limitation part).

: I have careful looked into your point, unfortunately I do not see the particular point. From Table 6, 117 of 1,200 cases have OF+/DCIP+/ or OF+ and DCIP+.

: In total, 97 cases of OF+; 79 case A2A, 1 case EE, 15 cases EA, 1 case A2A Barts H, and 1 case A2A with abnormal Hb.

: another 4 cases of A2A have OF- but DCIP+.

: Therefore, all OF positives have presented some form of Hb disdorder.

: 30 cases of alpha thalassemia 1 (SEA deletion), 29 cases have A2A (Hb A2<3.5%), and one case has A2A Bart’s H.

: However, if I missed the point please kindly let me know.

2.The author should demonstrate the “magnitude of the relation between thalassemia and the same-tribe grandparents” by using other statistical methods, for example, the logistic regression to show odds ratio (OR) and 95% CI.

: Thank you, we have used logistic regression to see the magnitude of having OF+ or DCIP+ or positive both OF and DCIP in different models. We have added the information into the result (Page 18-19) and discussion sections.

Thank you,

TK
