# Supplementary material for: Epidemiology of thalassemia among the hill tribe population in Thailand
Source: PLoS One. 2021 Feb 11;16(2):e0246736. doi: 10.1371/journal.pone.0246736 (PMC7877639; doi:10.1371/journal.pone.0246736)
Supplement: Attachment — Submitted filename: Reviewer attachment.docx [file pone.0246736.s006.docx]

This is a large epidemiologic study of thalassemia among the hill tribe population. The author showed differences in the prevalence of thalassemia and hemoglobinopathies among different tribes. The author demonstrated the relation between positive OF tests and the same-tribe grandparents. These findings reflected the association between consanguineous marriage and thalassemia disease. The author has addressed all comments. I have no further comment.
